# Hourly rainfall data from rain gauge networks and weather radar up to 2020 across the Hawaiian Islands

**DOI:** 10.1038/s41597-022-01430-2

**Published:** 2022-06-14

**Authors:** Yu-Fen Huang, Maxime Gayte, Yinphan Tsang, Ryan J. Longman, Alison D. Nugent, Kevin Kodama, Mathew P. Lucas, Thomas W. Giambelluca

**Affiliations:** 1 Natural Resources and Environmental Management, University of Hawaiʻi at Mānoa, Honolulu, HI USA; 2 East-West Center, Honolulu, HI USA; 3 Department of Atmospheric Sciences, University of Hawaiʻi at Mānoa, Honolulu, HI USA; 4 National Weather Service, Honolulu, HI USA; 5 Water Resources Research Center, University of Hawaiʻi at Mānoa, Honolulu, HI USA; 6 Department of Geography and Environment, University of Hawaiʻi at Mānoa, Honolulu, HI USA

**Keywords:** Atmospheric science, Hydrology

## Abstract

With increasing needs for understanding historic climatic events and assessing changes in extreme weather to support natural hazard planning and infrastructure design, it is vital to have an accurate long-term hourly rainfall dataset. In Hawaiʻi, annual, monthly, and daily gauge data have been well-compiled and are accessible. Here, we compiled hourly rainfall data from both gauges and radars. We arranged the metadata from various data sources, acquired data, and applied quality control to each gauge dataset. In addition, we compiled and provided hourly radar rainfall, and filtered out areas with low confidence (larger error). This paper provides (1) a summary of available hourly data from various observation networks, (2) 293-gauge rainfall data from their installation date to the end of 2020, and (3) a 5-year 0.005° by 0.005° (~250 × 250 *m*^2^) gridded radar rainfall dataset between 2016 and 2020 across the Hawaiian Islands.

## Abstract (summary)

**Table:** 

Measurement(s)	hourly rainfall
Technology Type(s)	weather station and weather radar
Sample Characteristic - Environment	water-based rainfall
Sample Characteristic - Location	State of Hawai'i

## Background & Summary

Flood related studies (e.g., hydrological modelling, impacts on flood intensity under changing climate, heavy rainfall and flood warning, and flood mitigation planning) require high spatial and temporal resolution rainfall data. In Hawaiʻi, among all the natural hazards, flooding causes the most property damage and frequently threatens residents’ lives^1^. Flash floods, in particular, can occur suddenly (<1 day) when a heavy rainfall event happens within a small watershed (<20 km^2^). Previous studies showed that using a limited number of rain gauges (e.g., two gauges) to simulate hydrological response produces unsatisfying results. This suggests that more measurements with high spatial resolution (<1 km) rainfall data provides improved depictions of hydrological response in Hawaiian watersheds^2,3^. In addition, with the growing focus on extreme weather driven by climate change, high temporal resolution (hourly or sub-hourly) rainfall data is becoming critical to study the intensity of rainfall and flooding. Hence, an hourly gridded rainfall dataset is essential in studying the impact of extreme weather in Hawaiʻi.

Current rainfall data in Hawaiʻi includes gauge rainfall products to the highest temporal resolution, daily, and radar hourly accumulated rainfall products to the finest spatial resolution, 0.24 × 1.5 *km*^2^ with Hydrologic Rainfall Analysis Project (HRAP) grid. Previous efforts on gauge rainfall involves compiling and mapping annual^4^, monthly^5–7^, and daily^8–10^ rainfall data. At the highest temporal resolution (daily) in the literature, Longman *et al*.^9^ published a dataset of daily gauge rainfall between 1990 and 2014 across the Hawaiian Islands. Longman *et al*.^8^ then extended their endeavors to map gridded daily rainfall. In addition to point rainfall measurement, weather radar (radar) (e.g., Next-Generation Radar, NEXRAD) provides high temporal-resolution with spatial information and is often used for flood modelling and forecasting in the contiguous United States (CONUS). There are four NEXRAD radars in Hawaiʻi, and public radar products include Level II^11^ and Level III^12^. The level II data consists of base reflectivity, while the level III data contains processed products, including hourly precipitation.

The existing rainfall data in Hawaiʻi is insufficient or difficult to apply to flood related studies. At present, although hourly rainfall is measured by gauges, efforts are still required before applying rain gauge measurement to any study: (1) the stations distribute among different networks and data stewards, and some of them cannot be accessed online directly; and (2) a quality-controlled dataset of hourly rainfall does not exist in most the gauge networks. The challenges and drawbacks of applying NEXRAD Level III rainfall data to hydrological studies include: (1) the non-rectangular HRAP grid of the rainfall dataset cannot be used directly to most of hydrological models; (2) the finest spatial resolution of the rainfall product is still considered coarse at one dimension (>1 km); (3) the confidence of radar and its products’ performance in Hawaiʻi are unknown; and (4) the parameters applied to the rain rate calculation are unclear. All of these hinder studies of radar rainfall in Hawaiʻi.

The goal of this paper is to describe the availability of hourly rainfall data in Hawaiʻi and provide: (1) rainfall data of 293 gauges from their installation date (the earliest, 1962) to 2020; and (2) a 0.005° by 0.005° ($\sim 250\times 250\;{m}^{2}$) gridded radar rainfall dataset between 2016 and 2020 under a level of confidence. For hourly gauge rainfall, we acquired all the data, applied quality controls (QCs), and compiled the metadata. For radar rainfall, we produced the rainfall product by converting raw radar reflectivity into hourly rainfall using the Lidar Radar Open Software Environment (LROSE)^13^. The paper is organized as follows: Methods—rainfall networks, step-by-step method for gauge rainfall QC, and the process to derive radar rainfall; Data records—an overview of the data files and their formats; and Technical Validations—comparing hourly radar rainfall with hourly gauge rainfall and things to be concerned when using the data.

## Methods

### Rain gauge networks

To compile a comprehensive Hawaiʻi-wide hourly rainfall dataset, we first identified and acquired data from all rain gauge networks. Based on the daily rain gauge networks between 1990 and 2014 in Longman *et al*.^9^, we extended the work to hourly rainfall data within all listed rain gauge networks and to the end of 2020. Additional networks and data repositories were explored and supplemented through this effort. The hourly rainfall data were assembled through several national and international online data repositories and networks (Table 1). For Hawaiʻi-only networks, data typically can only be obtained from project principal investigators (PIs) or data managers. Detailed information of nine out of 12 networks can be found in Longman *et al*.^9^ under the section, Data Records, with one network, Ua-Hydro Net, updated from Hydro Net. Besides the updated Ua-Hydro Net, three additional networks were identified and added to the data compilation in this study:

1. Ua-Hydro Net
  The Ua-Hydro Net (“ua” means “rain” in Hawaiian language) includes Ua Net and Hydro Net, maintained by Pacific Region Headquarters of National Weather Service (NWS). The gauges in Hydro Net (see Longman *et al*.^9^ for more detail) were gradually discontinued or converted to Ua Net beginning in mid-2015. Moreover, most of Ua Net data within the latest seven days can be retrieved from the Hydrometeorological Automated Data System (HADS).
2. Cooperative Observer Program Version 2
  The Cooperative Observer Program version 2 (COOPV2) has hourly gauge rainfall from 1940 to the present. These stations, nearly all of which were part of Hourly Precipitation Data (HPD) version 1 (a.k.a., DSI-3240), were gradually upgraded from paper punch tape data recording systems to a modern electronic data logger system from 2004–2013. Additionally, certain QCs have been applied to COOPV2 dataset^14^. Sixty-five stations recording rainfall data at 15-minute intervals were identified across the Hawaiian Islands in this network, with some of them co-located with Ua-Hydro Net stations. Data for these stations can be obtained through the National Centers for Environmental Information (NCEI) database (https://www.ncei.noaa.gov/data/coop-hourly-precipitation/v2/access/).
3. Hydrometeorological Automated Data System
  The Hydrometeorological Automated Data System (HADS) is a real-time data acquisition and data distribution system operated by the NWS. The HADS system acquires raw observations from Geostationary Operational Environmental Satellites (GOES) Data Collection Platforms (DCPs). Most of the data in Hawaiʻi acquired and processed by HADS come from the DCPs owned and/or operated by the NWS, Western Regional Climate Center (WRCC), Water Resources Division of the U.S. Geological Survey (USGS), and numerous local agencies and state departments of natural resources.
4. National Centers for Environmental Information Integrated Surface Database

**Table 1:** Rainfall Networks in Hawaiʻi.

Network	Spatial-Extent	# of stations	Link to the network/project description
HIPPNET^30^	Hawaiʻi-only	8	Personal contact; HIPPNET: http://www.hippnet.hawaii.edu/
Ua-Hydro Net	Hawaiʻi-only	82	Personal contact; Hydro Net: https://www.weather.gov/hfo/hydronet
UHM Lab	Hawaiʻi-only	39	Personal contact; Ecohydrology Lab: https://sites.google.com/a/hawaii.edu/ecohydrology_lab/; Tsang Stream Lab: https://tsangstreamlab.org/
ASOS	National	13	https://www.faa.gov/air_traffic/weather/asos/
HADS	National	127	https://hads.ncep.noaa.gov/
NCEI ISD^18^	National	12	https://www.ncei.noaa.gov/products/land-based-station/integrated-surface-database
NREL	National	1	https://midcdmz.nrel.gov/
RAWS	National	54	https://raws.dri.edu/
SCAN^31^	National	6	https://www.nrcs.usda.gov/wps/portal/wcc/home/aboutUs/monitoringPrograms/automatedSoilClimateMonitoring/
USCRN	National	2	https://www.ncei.noaa.gov/products/land-based-station/us-climate-reference-network
USGS^32^	National	33	https://waterdata.usgs.gov/nwis
COOPV2^14^	International	67	https://www.ncei.noaa.gov/access/metadata/landing-page/bin/iso?id = gov.noaa.ncdc:C00988

Note that some stations are cross listed by several networks in the # of station. The network, UHM Lab, includes the Ecohydrology Laboratory and Tsang Stream Laboratory. The Ecohydrology Lab is led by Dr. Thomas Giambelluca and has multiple networks, i.e., HaleNet, Little HaleNet, HavoNet, and CraterNet. The Tsang Stream Lab is led by Dr. Yinphan Tsang and has one station at Lyon Arboretum.

The NCEI Integrated Surface Database (ISD) is a global database that consists of hourly and synoptic surface observations compiled from numerous sources into a certain format and includes various meteorological parameters. Additionally, the ISD Lite, an advanced product of ISD, is a subset of the full ISD containing eight common surface parameters (air temperature, dew point temperature, sea level pressure, wind direction and wind speed, total cloud cover, hourly rainfall, and six-hour accumulated rainfall) in a fixed-width format free of duplicate values. Currently, there are 14 active NCEI ISD Lite stations updated daily in the NCEI ISD lite database for Hawaiʻi. Data can be obtained through the NCEI Data Access app (https://www.ncei.noaa.gov/products/land-based-station/integrated-surface-database).

### Radar networks

To derive radar quantitative precipitation estimates (QPEs), we use the raw radar reflectivity from NEXRAD LEVEL II dataset^11^ on Kauaʻi (PHKI), Molokaʻi (PHMO), and Hawaiʻi Island (PHKM and PHWA). Additionally, we also include radiosonde soundings on Kauaʻi and Hawaiʻi Island for radar QPE. We downloaded the sounding data from the atmospheric sounding website hosted by the Department of Atmospheric Science, University of Wyoming (http://weather.uwyo.edu/upperair/sounding.html). We use the Shuttle Radar Topography Mission (SRTM) 30-m resolution digital elevation data from the NCASA space shuttle STS-99 mission^15^ to calculate the beam blockage.

### Rain gauge data

**Fig. 1:**
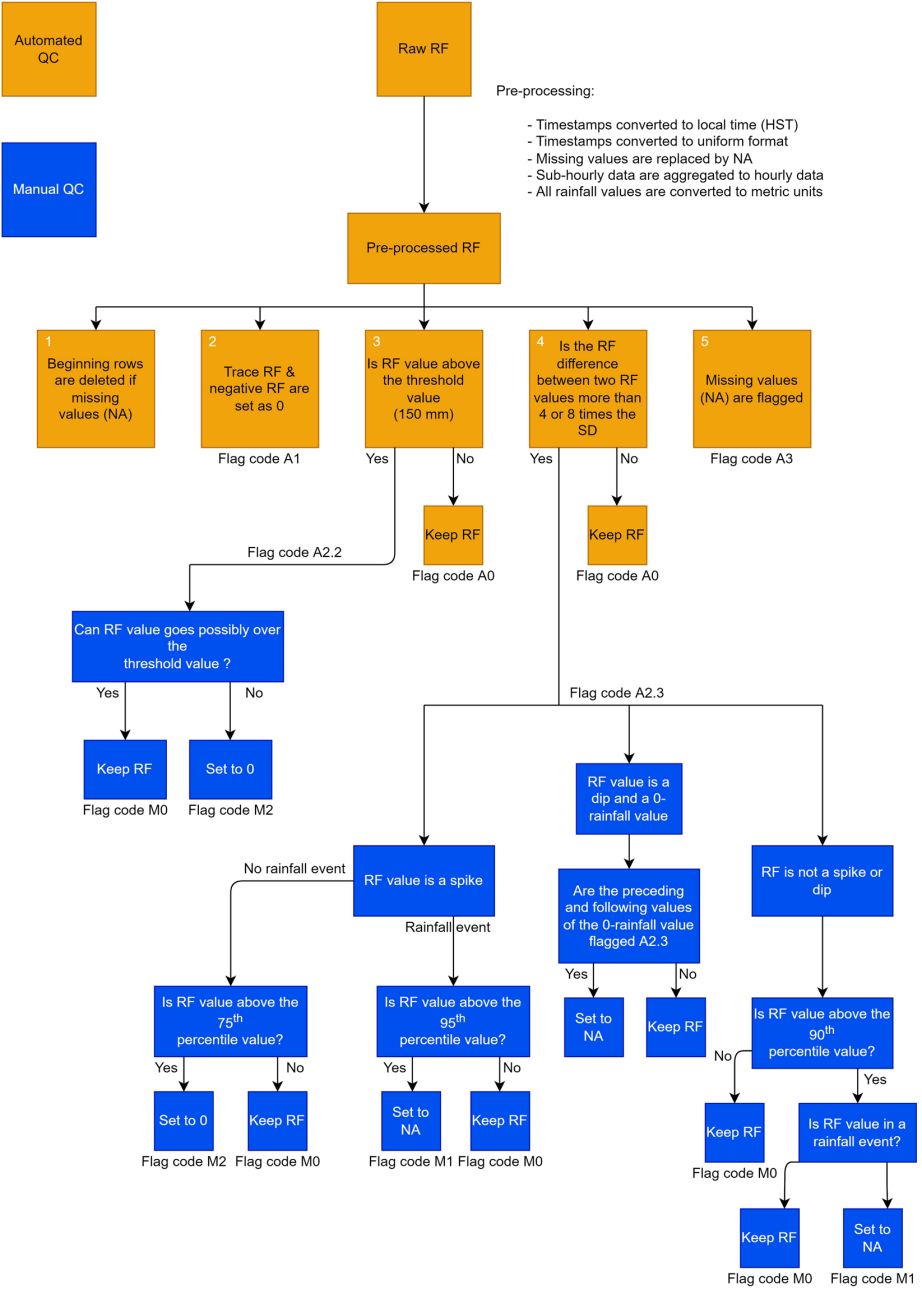
The flowchart of the quality control process. Yellow boxes are the automated flagging workflow and blue boxes are the manual flagging workflow.

We applied Quality Assessment and Quality Control (QAQC) to ensure a level of data homogeneity. Raw rainfall data acquired from various sources are subject to different formats and a wide variety of potential errors. Different networks have their own data format, and errors can occur during the data management chain of collecting, processing, transferring, and storing^16^. The World Meteorological Organization (WMO) developed a set of procedures for rescuing and quality controlling data^17^. Before conducting any scientific analysis, rainfall data must undergo a strict set of QAQC procedures to minimize the proportion of errors in the data. The data provided here were screened through an automated and a manual QC process (Fig. 1). We summarized the WMO QC steps and the adapted QC steps in Table 2. In addition, we compiled metadata for all gauges including their coordinates, record period, sources, and flags for the gauges with potentially unreliable data. We updated the latest coordinates in the metadata if the gauge was relocated yet retained the same gauge ID to be consistent with the source agencies.

**Table 2:** World Meteorological Organization (WMO) recommended climatological variables standard quality control (QC) steps and adapted QC steps in this paper. NA: missing value.

WMO recommended QC steps	Adapted QC steps in this paper
Format test (remove repeated observations, impossible format codes, impossible dates, and blank fields, etc.)	Pre-processing check (date and time format, rainfall unit, missing values set as NA)
Completeness test (flag any absence of expected observation)	Provide completeness of the data for each rainfall gauge
Tolerance test (set upper and/or lower limits)	Threshold value test (>150 mm)
Temporal consistency test (two times the standard deviation)	Standard deviation test (four to eight times depending on the number of NA before and after)

**Fig. 2:**
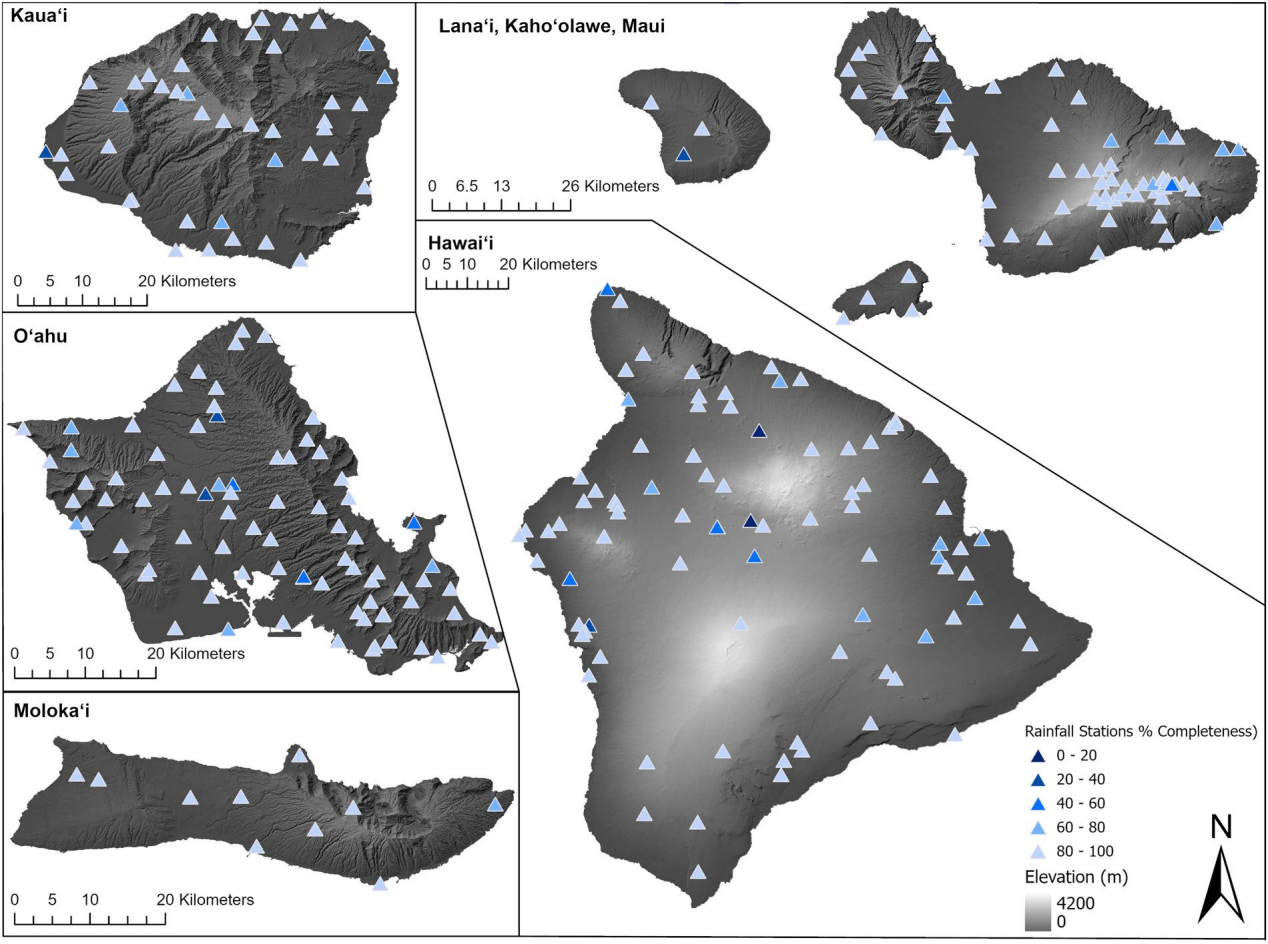
The location of the rain gauge and their completeness. Showing the locations of hourly rain gauges (triangles) and their completeness between the start-logging date of the gauge and December 31, 2020 (%; the color in triangles). The shaded of each island is the elevation (m).

Prior to conducting QC, a set of pre-processing protocols are applied to 293 gauges (Fig. 2).

1. All time stamps are converted to local time (Hawaiian Standard Time (HST), UTC-10).
2. Time stamps are converted to uniform format.
3. Missing values are replaced by NA.
4. Accumulated rainfall is changed to rain rate.
5. Hourly rainfall is aggregated by rain rate.
6. All rainfall values are converted to metric units (e.g., millimeters).
7. All stations were plotted using Geographic Information System (GIS) software to verify land-based locations. When two (or more) stations were co-located between NCEI IDS, COOPV2, and Ua-Hydro Net, we checked to see if they are the same stations included in multiple networks based on the Historical Observing Metadata Repository from NCEI (https://www.ncdc.noaa.gov/homr/). Once the station is confirmed, rainfall records of different periods were combined into one file. When periods of records overlapped, the records of the Ua-Hydro Net were prioritized to be kept in the file.

There are three main processes in the QC procedure—automated QC, manual QC (Fig. 1), and consistency with neighbor gauges. Once a common format of records is established, the following set of automated QC is applied. During these two processes, “flag codes” were given to the values detected by the QC criteria. A description of the automated QC process is given below, and flag codes are listed in Table 3.

1. Identify the beginning of the records. Delete the missing values (NA) at the beginning of the records.
2. Trace precipitation (<0.254 mm)^18^ or negative rainfall are set to 0 and flagged as A1.
3. Threshold test, flagged as A2.2:
  Rainfall values above 150 mm/hr^17^ are flagged.
4. Variability test, flagged as A2.3:
  If an hour before and after includes no missing values and the inspecting rainfall value varies more than eight times of standard deviation of gauge records (modified from WMO QAQC procedures^17^), the rainfall value is flagged.
  If an hour before or/and after the inspecting value includes missing value(s), the missing value(s) are assumed to be 0. If the inspecting rainfall value varies more than four times of standard deviation of gauge records, the rainfall value is flagged.
5. Missing value (NA) flagged as A3.
  After each file goes through the automated QC (steps 1 to 5), a manual QC (steps 6 to 7) was conducted to investigate and determine if the flagged rainfall values should be accepted, corrected, or deleted. A detailed description of the manual flag codes is given in Table 4.

**Table 3:** Automated QC flag codes and their definitions.

Automated Flag code	Definition
**A0**	Original data
**A1**	Trace precipitation set to 0
**A2.2**	Failed threshold test; need manual check
**A2.3**	Failed variability test; need manual check
**A3**	Missing data

**Table 4:** Manual QC flag codes and their definitions.

Manual Flag Code	Definition
**M0**	Kept original data
**M1**	Replaced by NA
**M2**	Replaced by 0
**(empty)**	No manual actions were made
6. Manual inspection of threshold flag A2.2:
  If the rainfall value occurs in a very extreme event, according to the verified information from NWS and the climatology of gauge, we referred the rainfall amount to the surrounding gauges and the entire records of the gauge to confirm that the rainfall value could possibly go over the defined threshold of 150 mm/hr.
7. Manual inspection of variability flag, A2.3:
  If the rainfall value is a spike (--˄--) in the time series
    and there is no rainfall event (i.e., no rain recorded within six hours before or after the flagged rainfall value hour), the rainfall value is accepted if it is under the 75th percentile of the entire dataset. For rainfall values above the 75th percentile, the values are set to 0 and flagged as M2. The threshold here was rationalized by the characteristics of locally convective rainfall—the variance during the no rainfall event should be lower than the variance of a larger and longer rainfall event (i.e., rain recorded within six hours).
    and a rainfall event is detected (i.e., rain recorded within six hours before or after the flagged rainfall value hour), the rainfall value is accepted if it is under the 95th percentile of the entire dataset. For rainfall values above the 95th percentile, the values are set to NA and flagged as M1. The 95th percentile was selected based on the fact that the 95th percentile value of an hourly rainfall dataset is large. Therefore, as large rainfall variations are expected when a rain event is observed, it is logical to compare the flagged value to a large value from the entire dataset.
  If the rainfall value is a dip (--v--) and is 0
    and rainfall records preceding and following the 0-rainfall value are flagged 2.3, the zero-rainfall value is set as NA and flagged as M1.
    and rainfall records preceding or following the zero-rainfall value are not flagged 2.3, the zero-rainfall value is accepted and flagged as M0.
  For the rainfall values that are not a spike (--˄--) or dip (--v--)
    and the value is lower than the 90th percentile value of the entire dataset, the value is accepted and flagged as M0.
    and the value is higher than the 90th percentile value of the entire dataset:
      values within a rainfall event (rain observed six hours before or after the flagged rainfall value) are accepted and flagged as M0.
      values that are not in a rainfall event (no rain observed six hours before or after the flagged rainfall value) are compared with satellite data, Tropical Rainfall Measuring Mission (TRMM)^19^, which provides 3-hour rainfall data for Hawaiʻi:
        if rain is observed with satellite data, the value is accepted and flagged as M0.
        if no rain is observed with satellite data, the value is set as NA and flagged as M1.

After flagging on hourly rainfall data of each gauge, we inspected the consistency of the gauge record and applied the correlation coefficient to the rainfall of each gauge and its nearest three (neighbor) gauges within a 6-km radius to examine any underreporting issues, based on the aggregated rainfall at a daily scale. We used three different flags to mark gauges including “Not Applicable”, “Warning”, and “Pass” (Table 5). Not Applicable was assigned when the nearest three gauges were outside of 6-km radius or when three neighbor gauges had no overlapping data periods for comparison. Warning was assigned when: (1) there were two neighbor gauges not applicable (missing due to the distance threshold or no overlapping data period), and the only remaining gauge had a correlation coefficient (R) less than 0.5; and (2) there were at most one not applicable neighbor gauge and at least two remaining gauges had R smaller than 0.5. Gauges that were not flagged as Not Applicable or Warning were considered passing the QC of neighbor gauge data comparison and marked as Pass. We included this flag under the column, CORR_FLAG in our metadata.

**Table 5:** Correlation coefficient flag codes and their definitions.

Correlation Coefficient flag code (CORR_FLAG)	Definition
**Not applicable**	The flag is not applicable on the gauge because there is not enough data to compare between its data and its neighbor gauges’ data.
**Warning**	The gauge needs to be used with caution because it has different daily rainfall pattern with its neighbor gauges.
**Pass**	The gauge is reliable because its data is consistent with its neighbor gauges’ data.

### Radar rainfall

We derived radar QPE by using the open sources software, Lidar Radar Open Software Environment (LROSE)^13^. LROSE is built on the legacy left by TITAN^20^ and developed by the National Center for Atmospheric Research (NCAR) to handle weather radar and lidar data. LROSE has very wide applications including beam blockage identification, converting radar variables into rain rate, and storm tracking, and is continuously updated. We use LROSE-cyclone, which was released in 2020^21^, to derive the QPE from the raw NEXRAD LEVEL II radar reflectivity.

There are three basic steps to obtain the radar QPE: (1) estimate precipitation rate aloft within the radar volume; (2) estimate the applicable rate at the surface; and (3) converse precipitation rate to precipitation depth over a certain period. Using LROSE, we estimated the precipitation rate at each radar grid by running the particle identification (PID) along with the nearest radiosonde soundings in time and space to estimate the 0 °C isotherm. The PID is derived by modified NCAR’s fuzzy-logic-based PID algorithm that can identify the particle types at each radar grid. After identifying particle types, we calculated the radar based QPE at the surface and applied the modified NCAR Hybrid method^22^ with localized parameters^23^ (Table 6) to calculate the precipitation rate at each grid point.

**Table 6:** Parameters and thresholds used in modified NCAR Hybrid method in Lidar Radar Open Software Environment (LROSE), where $Z{H}_{a}$ and $Z{H}_{b}$ are the coefficients of rain rate reflectivity relationship, dBZ is radar reflectivity, Kdp is specific differential phase, and Zdr is differential reflectivity.

Parameter	Value
${{\bf{ZH}}}_{{\bf{a}}}$	0.01
${{\bf{ZH}}}_{{\bf{b}}}$	0.83
**dBZ**	40
**Kdp (deg/km)**	0.3
**Zdr**	0.5

We applied the reflectivity-rain rate relationship, $Z=250{R}^{1.2}$. Thus, $rain\;rate=Z{H}_{a}\times reflectivit{y}^{Z{H}_{b}}$.

Prior to deriving the applicable precipitation rate at the surface, beam blockage was estimated at the lower elevation radar angles with the SRTM elevation data. The beam blockage algorithm considered standard atmospheric propagation effects and the convolution of the beam pattern with terrain features^22^. The algorithm produced a blocked fraction for each elevation angle. The beam blockage is crucial to derive QPE in Hawaiʻi because of the mountainous terrain. If it is blocked, then the remaining unblocked angle would be used for the precipitation estimation. The precipitation rate in polar coordinates at the surface then inherits the lowest elevation angles with the following criteria: (1) the signal to noise ratio (SNR) <5 dBZ; (2) beam blockage < = 25%; (3) PID does not show clutter, insects or second trip; and (4) the precipitation rate is not missing at the grid. The surface precipitation rate is then converted into Cartesian coordinates. The QPE was estimated by computing precipitation depth from the rate at the original time interval and summing the precipitation depth over time for accumulation periods (in this study, hourly), and was then converted into Cartesian grids. Finally, the precipitation rates from individual radars (if multiple radars were included) were integrated by taking the maximum value.

### Validation of radar rainfall

For this study we selected 18 severe rainfall events (Table 7) from the NCEI Storm Events Database (https://www.ncdc.noaa.gov/stormevents/). The events represent several different types of atmospheric disturbances across Hawaiian Islands including: three tropical cyclones, two cold fronts, seven upper-level troughs, two Kona lows, and four examples of mixing both Kona low and upper-level trough^24–26^. The hourly radar rainfall was validated with the quality-controlled gauge rainfall at each gauge location except for the gauges with correlation coefficients flagged as “Warning”. We defined the validation period of each event as low or no rainfall before and after the reported event time. For each event, the rain gauges for validating hourly radar rainfall must contain more than 80% of hourly rainfall records and the number of gauges can be slightly (<10) different. For each event, we applied four error matrices, including bias (BIAS), root mean square errors (RMSE), and their normalized values (nBIAS and nRMSE). BIAS indicates if radar rainfall is overall under- or over-estimated compared to the gauge rainfall, while RMSE quantifies the errors accumulated during the study periods and penalizes the larger error. To ensure that BIAS and RMSE are comparable among gauges and can be interpolated for creating masks of radar rainfall, the relative error matrices, nBIAS and nRMSE, were derived by dividing the range between event maximum and minimum rainfall of each gauge. In addition, we calculated the correlation coefficient (R) by combining all events. BIAS and RMSE indicate the accuracy at the gauges. nBIAS and nRMSE denote the spatial accuracy. The R value shows how well each gauge aligned with the radar estimate.

**Table 7:** Eighteen severe storm events selected for validation.

Episode ID (Storm Events Database)	Types	Validation period
**107196**	Tropical cyclone	2016/07/22 → 2016/07/26
**112867**	Cold front	2017/02/09 → 2017/02/13
**112880**	Mix of Kona low and upper-level trough	2017/02/27 → 2017/03/02
**120383**	Mix of Kona low and upper-level trough	2017/10/22 → 2017/10/25
**120841**	Upper-level trough	2017/11/10 → 2017/11/13
**122579**	Mix of Kona low and upper-level trough	2018/02/16 → 2018/02/20
**122592**	Upper-level trough	2018/02/23 → 2018/02/27
**123351**	Upper-level trough	2018/03/30 → 2018/04/02
**123655**	Upper-level trough	2018/04/11 → 2018/04/16
129101 & 129126^*^	Tropical cyclone	2018/08/24 → 2018/08/29
**130016**	Tropical cyclone	2018/09/11 → 2018/09/15
**131158**	Upper-level trough	2018/10/10 → 2018/10/13
**131163**	Kona low	2018/10/28 → 2018/11/01
**131895**	Cold front	2018/11/08 → 2018/11/12
**133682**	Upper-level trough	2019/02/12 → 2019/02/16
**137365**	Kona low	2019/06/23 → 2019/06/27
**141311**	Mix of Kona low and upper-level trough	2019/09/12 → 2019/09/16
**142475**	Upper-level trough	2019/10/09 → 2019/10/13

*Note: The event has two episode IDs because the Storm Event Database separated the episodes by the heavy rain directly influenced by the TC and the indirect heavy rain when the TC moved away.

### Mask of radar rainfall

**Fig. 3:**
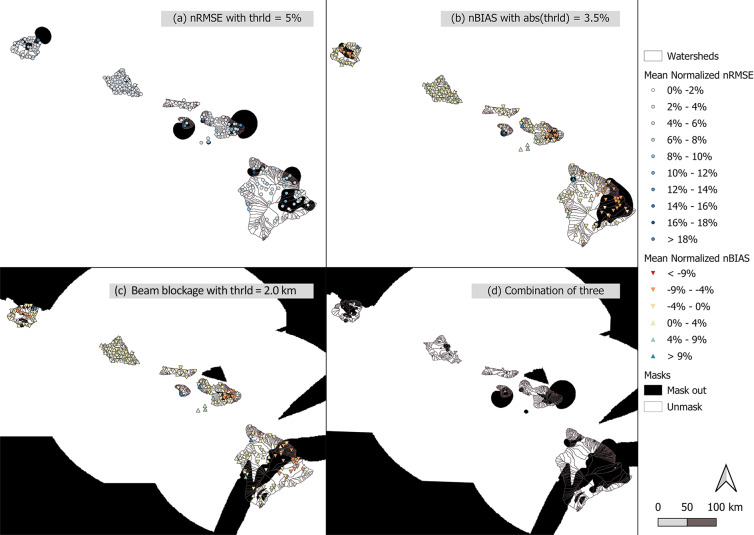
The masks for radar rainfall created by different indices. Showing the mask based on (**a**) normalized root mean square error (RMSE; circles) and its mask based on the threshold, RMSE >5% (black shaded); (**b**) normalized bias (BIAS; triangles) and its mask based on the threshold, |BIAS| >3.5% (black shaded); (**c**) normalized root mean square error (RMSE; circles) and the mask of beam blockage height at 2.0 km (black shaded); and (**d**) the mask that considered all threshold above (**a**–**c**; black shaded). Locations that are masked out do not have radar data provided.

To provide the gridded rainfall data with a level of confidence, we removed (masked out) areas with high uncertainties and bias by considering the high beam blockage areas, nBIAS, and nRMSE. Beam blockage is indicated by the higher beam blockage fraction or higher angle of the radar beam, which cause higher uncertainties and bias in reflectivity measurements. The beam blockage height is set for the lowest level that has the beam blockage fraction < = 25%. In addition, the nBIAS and nRMSE of radar rainfall against gauge rainfall were used to examine the confidence of radar measurement. Specifically, we examined the nBIAS and nRMSE of radar rainfall for the previously mentioned 18 weather events to ensure that the radar rainfall’s bias and error are not due to the type of event selected in this process. We filtered out the outliers of event nBIAS and nRMSE when Z-score >1 within the events at each gauge location. We then interpolated event-averaged nBIAS and nRMSE at each gauge location with inverse distance weight. Next, we applied thresholds: 5% for the interpolated nRMSE (Fig. 3a); 3.5% for the interpolated nBIAS (Fig. 3b); and 2.0 km for the beam blockage height (Fig. 3c). Lastly, we combined the masks to create a final mask (Fig. 3d). The mask is then applied to the radar rainfall to remove it from areas with higher uncertainties/errors. The remaining, unmasked, radar rainfall data are more reliable and have a higher level of confidence.

## Data Records

### Gauge rainfall

This dataset includes one metadata CSV file describing gauge data and location information (Table 8) and 293 comma-separated value (CSV) files of hourly gauge rainfall^27^. The metadata has been compiled in metadata_pub_V20220401.csv. The filename of each gauge is {FILENAME}.csv (FILENAME in metadata). Each hourly gauge rainfall csv file includes five columns: DateTime; RF_raw (raw hourly rainfall in millimeters); RF_mm (quality-controlled hourly rainfall in millimeters); flag_code_auto (the automated QC flag code); and flag_code_manual (the manual QC flag code). Note that the value, NA, means no measurement or uncertain rainfall amount. The metadata includes station information such as station name, coordinates, start and end date, data sources, etc. Table 8 shows the definition and unit of each column name in the metadata. The COMPLETENESS is provided so that users can gain a quick understanding of the percentage of how many hours have rainfall value compared to the entire observation period of the gauge. The hourly rainfall data starts from the earliest station record of hourly rainfall data and ends in the end of 2020 (i.e., December 31, 2020). One can refer to the SOURCES in the metadata to access rainfall data beyond 2020. The metadata and data records of this study dataset can be downloaded from the figshare electronic repository^27^. It is in the plan that gauge metadata and rainfall data will be updated to the Hawaiʻi Climate Data Portal (https://www.hawaii.edu/climate-data-portal/) prepared by Mclean *et al*.^28^ in the future.

**Table 8:** Metadata column names and their definition.

Column Name	Definition
**FILENAME**	Name of the file for download
**STATION_NAME**	Name of the station
**ISLAND**	The island of the station
**LAT**	Latitude (°)
**LON**	Longitude (°)
**ELEV_M**	Station elevation (m)
**DATE_START**	Start date of the data
**DATE_END**	End date of the data
**COMPLETENESS**	Completeness of the data between the start date and December 31, 2020 (%)
**SOURCES**	The source(s) of downloading data
**CORR_FLAG**	The flag for the consistency with its nearest 3 gauges (see Rain gauge networks)

### Radar rainfall

The masked hourly radar rainfall (2016–2020) is also provided on the same repository in figshare with the Network Common Data Form (NetCDF)^27^. The filename is “Hourly_radarRF_{YYYYMM}_masked.nc” (e.g., Hourly_radarRF_201601_masked.nc for masked radar rainfall in January 2016). The masked hourly radar rainfall is stacked into 60 NetCDF monthly files with three dimensions—time (UTC), latitude (degree), and longitude (degree). Each grid is by 0.005 degree (~ 250 m). The radar rainfall values on the grid with no radar, missing hours, and masked areas were filled as NaN with the same spatial dimension. There are 465 hours missing (260 hours in 2016, 25 hours in 2017, 12 hours in 2018, 137 hours in 2019, 31 hours in 2020) over the total 43,848 hours, and the data is 98.94% complete.

## Technical Validations

All hourly gauge rainfall data provided in this study are quality controlled and the radar rainfall was validated for 18 rainfall events. Out of a total of 293 stations, 215 stations remained active after December 31, 2020. The earliest data can be traced back to 1962 at Honolulu International Airport (911820–22521). Two new stations were established in 2020 at Pāpaʻikou well (PPWH1) and Waimea tank (WMTH1). The completeness ranges from 2.29% to 100% with a mean value of 87.04% (Fig. 2). The neighbor gauge comparison allows us to identify gauges with potentially unreliable data^29^. This QC process resulted in 225 Pass, 56 Not Applicable, and 12 Warning in our collective dataset. It successfully flagged the gauges that have inconsistent records with neighbor records to alert potential long period errors. For example, the gauge, 911905–22524, has many trace rainfall and potentially incorrect zero values (e.g., zero values at the station, while high rainfall values at other neighbor gauges) in the raw data. For these reasons, we recommend excluding the gauges flagged as Warning, or being extra cautious when using the data of these gauges.

**Fig. 4:**
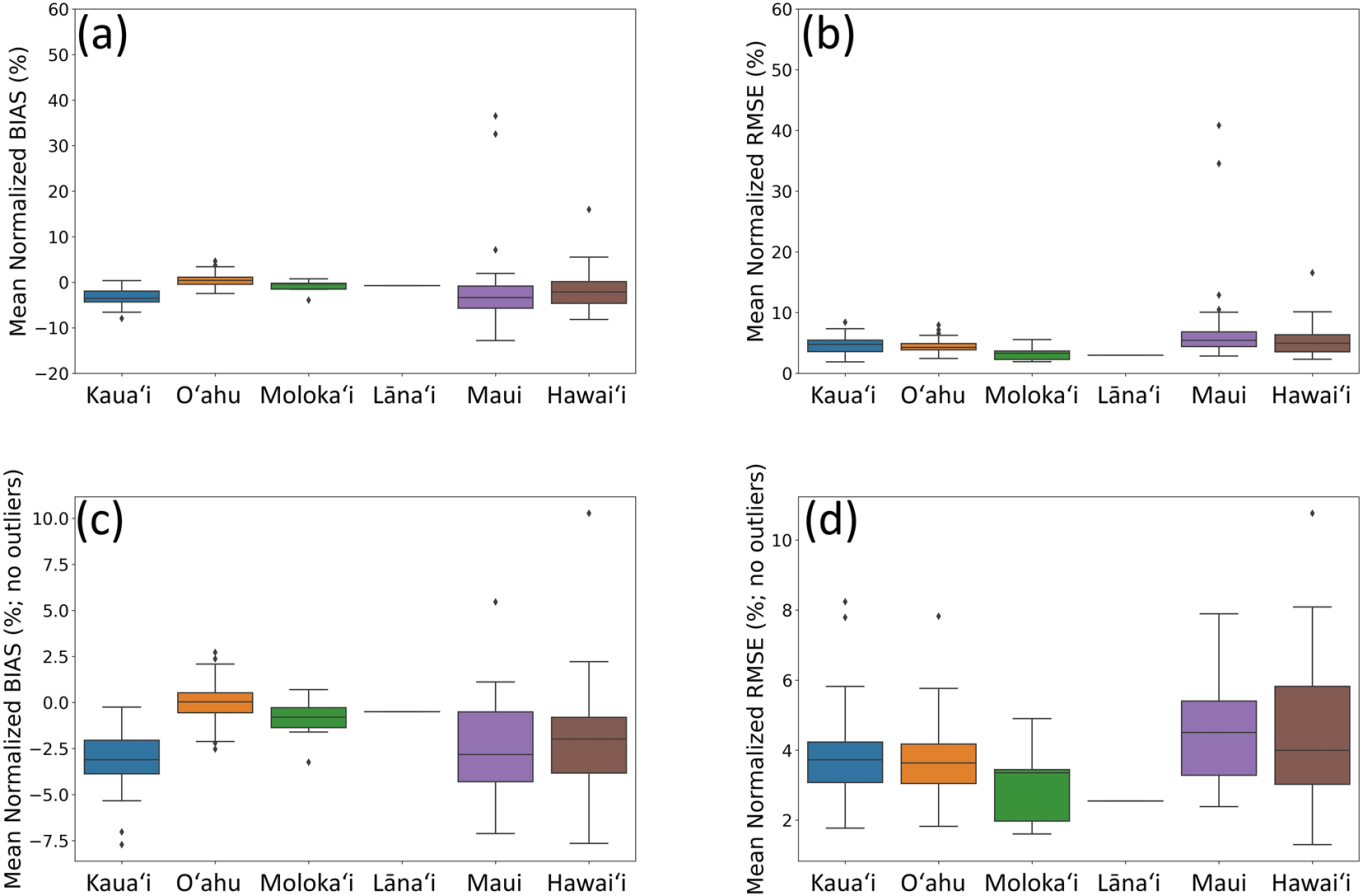
The validation boxplots. Showing the boxplots of event-averaged (**a**) normalized bias (nBIAS), (**b**) normalized root mean square errors (nRMSE), (**c**) nBIAS without outliers (Z-score < = 1), and (**d**) nRMSE without outliers (Z-score < = 1) of unmasked radar rainfall at each gauge location by the islands (n = 31, 71, 9, 1, 53, and 75 for Kauaʻi, Oʻahu, Molokaʻi, Lānaʻi, Maui, Hawaiʻi, respectively).

When validating the mean BIAS, RMSE, nBIAS, nRMSE, and correlation coefficient over all gauges are $-0.32$ mm, 0.61 mm, $-1.30 \%$, $5.23 \%$, and 0.60, respectively, before applying the mask. The hourly radar rainfall has better agreement with the hourly gauge rainfall on Oʻahu and Molokaʻi (Fig. 4). The radar rainfall often underestimates the rainfall on Kauaʻi, Maui, and Hawaiʻi compared to gauge rainfall (Fig. 4). When validating the remaining radar data against 153 gauges, the final masked-out radar rainfall has an increased confidence with mean BIAS, RMSE, nBIAS, nRMSE, and CORR as $-0.10$ mm, 0.43 mm, $-0.14 \%$, $4.43 \%$, and 0.67, respectively. The masked hourly radar rainfall has more confidence and reliability than the former unmasked data, thus, we provide the masked radar rainfall here.

## Data Availability

The R version 4.0.2 along with the R packages, dataRetrieval (v2.7.6), lubridate (v1.7.9.2), dplyr (v1.0.2), and data.table (v1.13.6) are used to download and quality control hourly gauge rainfall data. We used the 2020^17^ Lidar Radar Open Software Environment (LROSE) to derive hourly radar rainfall. The python version 3.7.10 along with the python packages, pandas (v1.3.5), numpy (v1.21.2), netCDF4 (v1.5.8), xarray (v0.19.0), and matplotlib (3.5.0) are used for the validation process.
