# Publisher Correction: Hourly rainfall data from rain gauge networks and weather radar up to 2020 across the Hawaiian Islands

**DOI:** 10.1038/s41597-022-01617-7

**Published:** 2022-08-18

**Authors:** Yu-Fen Huang, Maxime Gayte, Yinphan Tsang, Ryan J. Longman, Alison D. Nugent, Kevin Kodama, Mathew P. Lucas, Thomas W. Giambelluca

**Affiliations:** 1 Natural Resources and Environmental Management, University of Hawaiʻi at Mānoa, Honolulu, HI USA; 2 East-West Center, Honolulu, HI USA; 3 Department of Atmospheric Sciences, University of Hawaiʻi at Mānoa, Honolulu, HI USA; 4 National Weather Service, Honolulu, HI USA; 5 Water Resources Research Center, University of Hawaiʻi at Mānoa, Honolulu, HI USA; 6 Department of Geography and Environment, University of Hawaiʻi at Mānoa, Honolulu, HI USA

**Keywords:** Atmospheric science, Hydrology, Atmospheric science, Hydrology

Correction to: *Scientific Data* 10.1038/s41597-022-01430-2, published online 14 June 2022

In the original version of this Article apostrophe’s were used in place of ʻokina in Hawaiian words; one gauge name (Pāpaʻikou) was also misspelled.

In the original version of this Article a pointed upwards (--˄--) symbol was used for indicating a dip rainfall value instead of a downwards (--˅--) symbol.

In the original version of this Article reference 27 was missing “*et al*” in the author list.

These have been corrected in both the PDF and HTML versions of the Article.

